# Dr. Simon

**Published:** 1912-03

**Authors:** 


IN the February issue, we noted the death of a medical hero, Dr. Simon, of Zurich, who died from septic infection incurred in laboratory investigations. On Feb. 12, Dr. G. Armeauer of Christiana, Norway, a biologist and the discoverer of the lepra bacillus; long in charge of the leper hospital at Bergen, died. He was no less a hero because death was not due directly to the hazards of battle against disease. And here follows what is perhaps still greater heroism: Dr. Mary Baldwin, who had devoted her life to "mute, inglorious" service to the poor of New York, without hope of fame, beginning at a time when merely to be a woman and a doctor of medicine, betokened heroism, was found, a few weeks ago, old, sick and starving, in a wretched room on the Bowery. She was taken to the city hospital on Blackwell's Island, died Feb. 12, aged 76 and her body lay among the unclaimed pauper dead at the morgue. A PAUPER, mind you, this woman, who had given her life work to the people; who had donated technical skill who had repeatedly incurred risks of all sorts and had suffered physical exposure. Yet there is a comfortable, indoor form of philanthropy, requiring no expert training, for which fair, if not generous salaries are paid. One need not expect gratitude, nor the ability to make its gratitude practical from the class to which this professional sister devoted herself, but it does seem as if, from the vast amount of money spent in charity, a few dollars might have been spared to have given this charity worker, a death bed and a grave free from the inevitable if not entirely logical stigma of pauperism. The public owes a debt to such as this woman, a debt which is not paid by the routine relief afforded to the proletariat. But, let us not forget that the organized medical profession is receiving from its individual members, and by its legitimate earnings as a corporation, somewhere in the neighborhood of a third of a million dollars a year. Should we not set aside a small part of this for the care of worthy and unfortunate colleagues?

